# Diet-induced obesity causes peripheral and central ghrelin resistance by promoting inflammation

**DOI:** 10.1530/JOE-15-0139

**Published:** 2015-07

**Authors:** Farhana Naznin, Koji Toshinai, T M Zaved Waise, Cherl NamKoong, Abu Saleh Md Moin, Hideyuki Sakoda, Masamitsu Nakazato

**Affiliations:** 1 Division of Neurology, Respirology, Endocrinology and Metabolism, Department of Internal Medicine, Faculty of Medicine, University of Miyazaki, 5200 Kihara, Kiyotake, Miyazaki, 889-1692, Japan; 2 Department of Sports and Fitness, Faculty of Wellness, Shigakkan University, 55 Nakoyama, Yokone, Obu, 474-8651, Japan; 3 AMED-CREST, Agency for Medical Research and Development, 1-7-1 Otemachi, Chiyoda-ku, Tokyo, 100-0004, Japan

**Keywords:** ghrelin, diet-induced obesity, nodose ganglion, vagus nerve, inflammation

## Abstract

Ghrelin, a stomach-derived orexigenic peptide, transmits starvation signals to the hypothalamus via the vagus afferent nerve. Peripheral administration of ghrelin does not induce food intake in high fat diet (HFD)-induced obese mice. We investigated whether this ghrelin resistance was caused by dysfunction of the vagus afferent pathway. Administration (s.c.) of ghrelin did not induce food intake, suppression of oxygen consumption, electrical activity of the vagal afferent nerve, phosphorylation of ERK2 and AMP-activated protein kinase alpha in the nodose ganglion, or Fos expression in hypothalamic arcuate nucleus of mice fed a HFD for 12 weeks. Administration of anti-ghrelin IgG did not induce suppression of food intake in HFD-fed mice. Expression levels of ghrelin receptor mRNA in the nodose ganglion and hypothalamus of HFD-fed mice were reduced. Inflammatory responses, including upregulation of macrophage/microglia markers and inflammatory cytokines, occurred in the nodose ganglion and hypothalamus of HFD-fed mice. A HFD blunted ghrelin signaling in the nodose ganglion via a mechanism involving *in situ* activation of inflammation. These results indicate that ghrelin resistance in the obese state may be caused by dysregulation of ghrelin signaling via the vagal afferent.

## Introduction

Control of food intake in the brain is regulated by the integration of both the neuronal and humoral signals from the periphery. A variety of sensory information derived from the gastrointestinal tract is transmitted to the nucleus of the tractus solitaries (NTS) in the medulla oblongata via the vagal afferent nerve, terminating in hypothalamic nuclei implicated in the control of feeding (Rinaman 2010). The nodose ganglion, located outside the jugular foramen, is a constellation of vagal afferent neurons that synthesize receptors for gut peptides that regulate feeding and energy homeostasis (Zhuo *et al*. 1997, Konturek *et al*. 2004). These receptors are transported to afferent terminals in the gastrointestinal mucosa, which are more optimally positioned to monitor bioactive substances released from gastrointestinal enteroendocrine cells. Nodose ganglion neurons are pseudounipolar neurons with two axons running towards the visceral organs and the NTS.

Ghrelin, a peptide primarily produced in the stomach, stimulates feeding (Kojima *et al*. 1999, Nakazato *et al*. 2001). Ghrelin exists in two major forms, *n*-octanoyl-modified ghrelin and des-acyl-ghrelin (a non-acylated form of ghrelin). The growth hormone secretagogue receptor (GHSR), also known as the ghrelin receptor, is synthesized in vagal afferent neurons and transported to the stomach by axonal transport (Date *et al*. 2002). Ghrelin binds to this receptor and suppresses the electrical activity of the gastric vagal afferent. This information is transmitted to the NTS and relayed via the noradrenergic pathway to the hypothalamic neurons expressing orexigenic neuropeptides, neuropeptide Y (NPY) and agouti-related peptide (AgRP) (Date *et al*. 2006). Diet-induced obesity (DIO) causes resistance to central administration of ghrelin by suppressing expression of the ghrelin receptor in NPY/AgRP neurons (Briggs *et al*. 2010). Peripheral administration of ghrelin also failed to induce feeding in DIO mice (Briggs *et al*. 2010). However, the mechanism underlying this unresponsiveness remains to be demonstrated.

Immune-cell-mediated tissue inflammation in the adipose tissue, liver, and skeletal muscle plays a critical role in the development of obesity and insulin resistance (Hotamisligil *et al*. 1993, Schenk *et al*. 2008). Obesity-associated inflammation, including enhanced expression of interleukin 1 beta (IL1β), tumor necrosis factor alpha (TNFα), and IL6 in the hypothalamus, was first reported in 2005 (De Souza *et al*. 2005), and many investigators have since replicated this finding (Cai & Liu 2011, Thaler *et al*. 2013). DIO attenuated both sensitivities of vagal afferents to the satiety mediators and membrane excitability of vagal afferents (Daly *et al*. 2011), indicating that the development of obesity may be related to impairments in the vagal afferent system.

The aim of the present study was to investigate whether DIO-induced inflammatory responses in the nodose ganglion mediate ghrelin resistance in the vagal afferent system. We studied ghrelin's effects on feeding, energy consumption, electrical activation of the vagus afferent, and neuronal activation in the hypothalamus of DIO mice fed a high fat diet (HFD) for 12 weeks. Expression of the ghrelin receptor in both the nodose ganglion and hypothalamus were downregulated in HFD-fed mice. We also investigated inflammation in the nodose ganglion and hypothalamus by performing immunohistochemistry of macrophages/microglia and mRNA expression profiling of inflammatory cytokines.

## Materials and methods

### Animals

Male C57BL/6J mice (6-week-old, 20–21 g, Charles River Laboratories, Yokohama, Japan) were maintained in individual cages under controlled temperature (21–23 °C) and light (light on: 0800–2000 h) conditions. They were maintained on either chow diet (CD, 12.3% fat, 59.2% carbohydrate, 28.5% protein, and 14.2 kJ/g; CLEA Rodent Diet CE-2, CLEA Japan, Inc., Tokyo, Japan) or HFD (60% fat, 20% carbohydrate, 20% protein, and 21.9 kJ/g; no. D12492; Research Diets, New Brunswick, NJ, USA) with free access to food for 12 weeks. Cannulae were implanted i.c.v. into the lateral cerebral ventricle under anesthesia by i.p. injection of sodium pentobarbital (Abbot Laboratories). Only animals demonstrating progressive weight gain after the surgery were used in subsequent experiments. All animal experiments were approved by the Animal Care and Use Committee of University of Miyazaki.

### Characteristics of HFD-fed mice

Mice fed CD or HFD for 12 weeks (*n*=8/group) were fasted from 0900 to 1400 h, and then blood was collected by tail-prick. Blood glucose was measured with a glucometer (Terumo, Tokyo, Japan), and plasma insulin was measured using a mouse insulin EIA Kit (Morinaga Institute of Biological Science, Yokohama, Japan). For plasma ghrelin and leptin measurements, CD- or HFD-fed mice were deeply anesthetized with sodium pentobarbital, and blood samples were collected by cardiac puncture. Plasma ghrelin was measured using an active ghrelin ELISA Kit (Mitsubishi Chemical Medience, Tokyo, Japan; intra- and inter-assay precision coefficient of variation (CV) <10%, assay range 2.5–160 fmol/ml) and des-acyl ghrelin with a des-acyl ghrelin ELISA Kit (Mitsubishi Chemical Medience; intra- and inter-assay precision CV <10%, assay range 12.5–800 fmol/ml). Plasma leptin was measured using a mouse/rat leptin ELISA Kit (Morinaga Institute of Biological Science; intra- and inter-assay precision CV ≤10%, assay range 0.4–25.6 ng/ml). All samples were measured in duplicate. The amount of daily food intake was measured for 4 days before the administration experiments. Epididymal fat weight was measured at killing.

### Food intake experiments

Mice fed CD or HFD (*n*=6/group) for 11 weeks were transferred to single cages and maintained for 1 week, during which they were acclimatized by s.c. injections of saline once daily for 3 days. In the first experiment, mice (*n*=6/group) received subcutaneously administered ghrelin (60 nmol/kg body weight (BW); Peptide Institute, Osaka, Japan) or saline. In the second experiment, mice (*n*=6/group) received an i.c.v. injection of artificial cerebrospinal fluid (aCSF) or ghrelin (500 pmol). Administration was performed at 1000 h in both experiments, and 2-h food intake was measured. In the third experiment, mice (*n*=6/group) received an i.c.v. injection of anti-ghrelin IgG (0.5 μg/2 μl aCSF) prepared elsewhere (Nakazato *et al*. 2001) or normal rabbit serum IgG (0.5 μg/2 μl aCSF) at 1800 h. In the fourth experiment, mice (*n*=6/group) received an i.p. injection of leptin (2 μg/g BW; Sigma–Aldrich) or saline at 2000 h. Dark-phase food intake was measured in the third and fourth experiments.

### Oxygen consumption

Mice fed CD or HFD (*n*=4/group) for 11 weeks were housed in a metabolic chamber (Shinfactory, Fukuoka, Japan) for 1 week. They were given s.c. injection of ghrelin (60 nmol/kg BW) or saline at 1000 h, and then returned to the chambers. Oxygen consumption was measured in an Oxymax (Columbus Instruments, Columbus, OH, USA) for 120 min. Mice were deprived of food during the measurement.

### Time-course of plasma ghrelin concentration after s.c. administration of ghrelin

Mice fed CD or HFD (*n*=3/group) were subcutaneously administered ghrelin (60 nmol/kg BW). Blood was taken from the tail vein 0, 15, 30, 60, and 120 min after administration and immediately collected into tubes containing disodium EDTA (1 g/l) with aprotinin (500 kIU/l) (Wako Pure Chemicals, Osaka, Japan). Plasma was mixed with 1 M HCl (10% of plasma volume). Ghrelin was measured using an active ghrelin ELISA Kit.

### Electrophysiology study

Multiunit neural discharge in gastric vagal afferent fibers was recorded extracellularly. CD- or HFD-fed mice were anesthetized by an i.p. injection of urethan (1 g/kg; Sigma–Aldrich). For electrophysiological studies, animals were anesthetized throughout the procedure. Standard methods of extracellular recording from vagal nerve filaments were used, as developed in our laboratory (Date *et al*. 2005). In brief, we placed filaments isolated from the gastric branch of the vagal trunk peripheral, cut under the diaphragm for recording of afferent nerve activity, on a pair of silver wire electrodes. Silver wire electrodes, connected through a Differential Extracellular Amplifier (ER-1; Cygnus Technology, Delaware Water Gap, PA, USA) to a PowerLab/8SP (ADInstruments, Melbourne, FL, Australia), were used to record neural activity. The number of spikes was calculated using the Labchart 7 Software (ADInstruments) with a rate meter. After 10 min recording of basal nerve discharges from the multiunit afferents, these nerve discharges were continually recorded for 15 min after s.c. administration of saline or ghrelin (60 nmol/kg BW; *n*=4/group) in CD- or HFD-fed mice. The total number of spikes for 15 min after administration was calculated.

### Fos expression

Mice (*n*=3/group) received an i.c.v. administration of ghrelin (500 pmol/2 μl aCSF), a s.c. administration of ghrelin (60 nmol/kg BW), or saline 90 min before transcardial perfusion with 4% paraformaldehyde. They were anesthetized with sodium pentobarbital and transcardially perfused with ice-cold heparinized 0.1 M phosphate buffer (PB, pH 7.4) for 20 min, and then with ice-cold 4% paraformaldehyde in PB for 20 min. The brain was removed and post-fixed overnight in the fixative solution containing 4% paraformaldehyde, and then cryoprotected in 0.1 M PB containing 20% sucrose. We cut 40-μm sections of the hypothalamus. Fos immunohistochemistry was performed using a method described elsewhere (Toshinai *et al*. 2003). Briefly, free-floating sections were incubated in 0.3% hydrogen peroxide for 10 min, blocked with 1% normal goat antiserum (Santa Cruz Biotechnology), and incubated in rabbit Fos antiserum (1:500 dilution, Santa Cruz Biotechnology) in 0.01 M PBS (pH 7.4) overnight at 4 °C with gentle agitation. Sections were then incubated in biotinylated goat anti-rabbit IgG (1:500 dilution, Vector Laboratories, Burlingame, CA, USA), and immunoreactivity was visualized using the avidin–biotin–peroxidase complex reaction method with diaminobenzimide (VECTASTAIN Elite Kit, Vector Laboratories). Images were captured on an OLYMPUS AX-7 microscope (Olympus). Fos-positive cells were automatically counted in the sections using a cell-counting program (Bio-Imaging Analysis System Lumina Vision, Tokyo, Japan).

### Real-time PCR

The nodose ganglion and hypothalamus (*n*=8–12/group) were removed from anesthetized CD- or HFD-fed mice. Total RNA was extracted with a RiboPure Kit (Ambion, Austin, TX, USA). RT-PCR was conducted on a LightCycler System (Roche Diagnostics) using SYBR Premix Ex Taq (2×) (Takara Bio, Shiga, Japan) and the following primer sets: mouse *Ghsr*, 5'-ATCACCTCTGGGTCTTGTTGCTG-3' and 5'-GCTGAATGGCTCATTGTAGTCCTG-3'; ionized calcium binding adapter molecule (*Iba1*), 5'-AGCTGCCTGTCTTAACCTGCATC-3' and 5'-TTCTGGGACCGTTCTCACACTTC-3'; Egf-like module-containing, mucin-like, hormone receptor-like 1 (*Emr1*), 5'-GAGATTGTGGAAGCATCCGAGAC-3' and 5'-GACTGTACCCACATGGCTGATGA-3'; *Il6*, 5'-CCACTTCACAAGTCGGAGGCTTA-3' and 5'-CCAGTTTGGTAGCATCCATCATTTC-3'; *Il1b*, 5'-TCCAGGATGAGGACATGAGCAC-3' and 5'-GAACGTCACACACCAGCAGGTTA-3'; *Tnf* *α*, 5'-TATGGCCCAGACCCTCACA-3' and 5'-GGAGTAGACAAGGTACAACCCATC-3'; Toll-like receptor 4 (*Tlr4*), 5'-GGAAGTTCACATAGCTGAATGAC-3' and 5'-CAAGGCATGTCCAGAAATGAGA-3'; *Tlr2*, 5'-TGTCTCCACAAGCGGGACTTC-3' and 5'-TTGCACCACTCGCTCCGTA-3'; *Tbp*, 5'-CATTCTCAAACTCTGACCACTGCAC-3' and 5'-CAGCCAAGATTCACGGTAGATACAA-3'; and *Gapdh*, 5'-TCAAGAAGGTGGTGAAGCAG-3' and 5'-TGGGAGTTGCTGTTGAAGTC-3'. The obtained values were normalized against that for *Gapdh* or *Tbp*, used as an internal control.

### Immunohistochemistry

Nodose ganglia and whole brains (*n*=4/group) were immersed in 4% paraformaldehyde/PB for 24 h at 4 °C, incubated for 24 h in PB containing 20% sucrose, quickly frozen on dry ice, and cut into 8-μm slices with a cryostat at −20 °C. Sections blocked for 5 min in protein-block serum-free solution (Dako, Carpinteria, CA, USA) were incubated overnight at 4 °C with rabbit anti-IBA1 (1:10 000; Wako Pure Chemicals), rat anti-CD11b (1:50; AbD Serotec, Oxford, UK), and rat anti-CD86 (1:100; Abcam, Cambridge, UK). Immunofluorescence was performed with a combination of Alexa Fluor 488-labeled anti-rabbit secondary antibody or Alexa Fluor 594-labeled anti-rat secondary antibody (both 1:400; Invitrogen). Images were captured on an OLYMPUS AX-7 fluorescence microscope (Olympus). Cells immunostained with IBA1, CD11b, or CD86 antibody were counted manually with Olympus cellSens Imaging Software (Olympus). Quantitation was performed in a blinded fashion.

### Western blotting

Mice (*n*=6/group) fed a CD or HFD for 12 weeks were anesthetized and received subcutaneous injections of ghrelin (60 nmol/kg BW) or saline. They were perfused with PB 60 min later for AMP-activated protein kinase alpha (AMPKα) measurement, or 120 min later for ERK1/2 measurement, then the nodose ganglion was isolated. Protein (10–20 μg) extracted from the nodose ganglion was separated on SDS–PAGE Tris–glycine gels (Mini-PROTEAN TGX Precast Gels, Bio-Rad) for 100 min at 75 V and transferred to nitrocellulose membrane. Membranes were blocked with 5% (w/v) non-fat dry milk and incubated with antibodies for phosphorylated Erk1/2 (Thr^202^/Thr^204^) (1:4000), Erk1/2 (1:4000), pAMPKα (1:2000), AMPKα (1:2000), or Gapdh (1:5000) (all five from Cell Signaling Technology Japan, Tokyo, Japan) in blocking buffer overnight at 4 °C. Membranes were then incubated with the corresponding secondary antibodies. For sequential analysis of membranes, bound antibodies were removed with stripping buffer (10% SDS, 1 M Tris–HCl, and pH 6.8) for 30 min at 55 °C. After washes, membranes were developed in ECL buffer (ImmunoStar LD, Wako Chemicals USA, Richmond, VA, USA) for 1 min. The chemiluminescent blots were visualized and imaged using a Syngene G: BOX iChemi XR System (Syngene, Cambridge, UK) and densitometry was performed on the lanes using the GeneTools Software (version 4.01; Syngene) to quantitate protein expression. Band intensities were normalized by calculating the respective ratios of the intensities of the bands of pERK to ERK or pAMPKα to AMPKα.

### Statistical analysis

Statistical analyses were performed by one- or two-way ANOVA followed by a Bonferroni's post-test for multiple comparisons, as appropriate. When two mean values were compared, analysis was performed by Mann–Whitney *U* test or Wilcoxon's or unpaired *t*-test. All data are expressed as means±s.e.m. *P*<0.05 was considered to be statistically significant.

## Results

### Characterization of HFD-fed mice

Table 1 shows characteristics and blood parameters in mice fed a HFD for 12 weeks. Food intake amount in HFD-fed mice was significantly lower than in CD-fed mice, whereas energy intake of HFD-fed mice (57.4 kJ) was significantly higher than that of CD-fed mice (46.5 kJ). Body weights and epididymal fat weights in HFD-fed mice were higher than those in CD-fed mice. HFD caused significant increases in fasting blood glucose, plasma insulin and leptin, and decreases in plasma ghrelin and des-acyl ghrelin.

### Ghrelin and leptin responses

**Figure 1:**
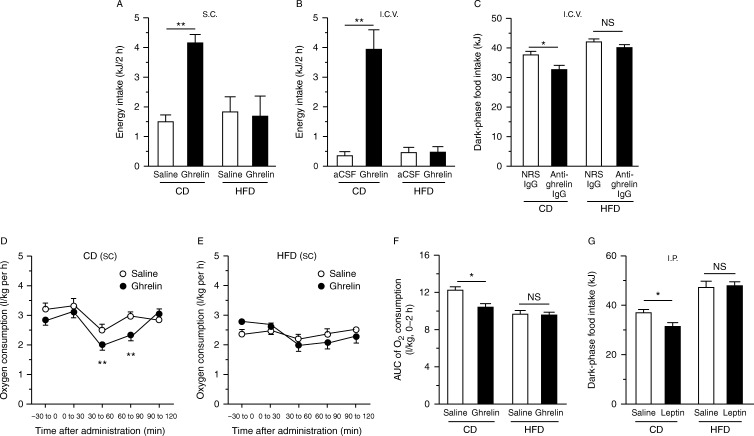
Effects of ghrelin on food intake (A, B and C), and oxygen consumption (D, E and F) of mice fed a CD or a HFD for 12 weeks. Two-hour food intake during the light phase in response to s.c. (A) or i.c.v. (B) administration of ghrelin, and dark-phase food intake (C) in response to i.c.v. administration of NRS IgG or anti-ghrelin IgG, in CD- or HFD-fed mice. Oxygen consumptions in CD- (D) or HFD-fed mice (E) subjected to s.c. ghrelin administration, and area under the curve (AUC) of oxygen consumption from 0 to 2 h after ghrelin administration (F). Dark-phase food intake of CD- or HFD-fed mice subjected to leptin administration (G). NS, not significant. Values are means±s.e.m. *P<0.05 and **P<0.01.

Both s.c. and i.c.v. administrations of ghrelin increased food intake in CD-fed mice, but not HFD-fed mice (Fig. 1A and B). Inversely, i.c.v. administration of anti-ghrelin IgG reduced dark-phase food intake in CD-fed mice, but not HFD-fed mice (Fig. 1C). S.c. administration of ghrelin reduced oxygen consumption in CD- but not HFD-fed mice (Fig. 1D, E and F). Leptin administration did not reduce food intake in HFD-fed mice (Fig. 1G).

### Time-course of plasma ghrelin concentration

**Figure 2:**
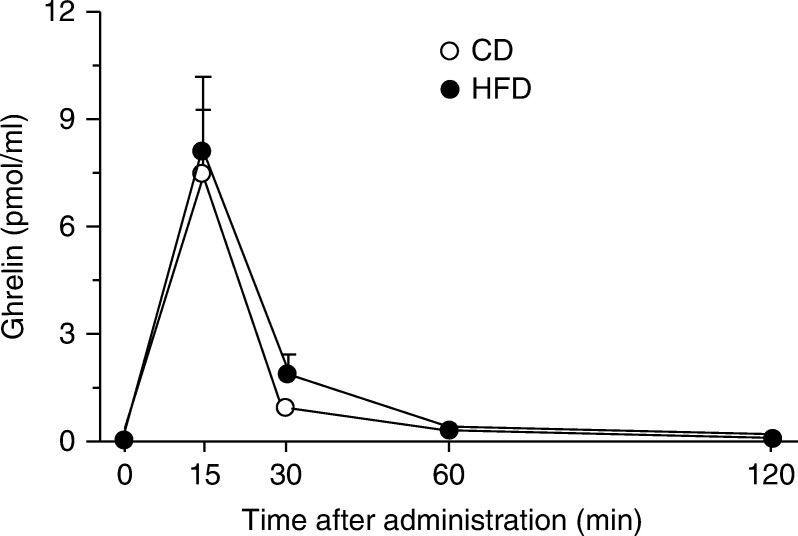
Time course of plasma ghrelin concentrations after s.c. administration to CD- or HFD-fed mice. Values are means±s.e.m.

We compared time courses of plasma concentrations of ghrelin administered subcutaneously to CD- or HFD-fed mice (Fig. 2). The time courses of plasma ghrelin disappearance were similar between the two groups.

### No effect of ghrelin on vagal afferent activity in HFD-fed mice

**Figure 3:**
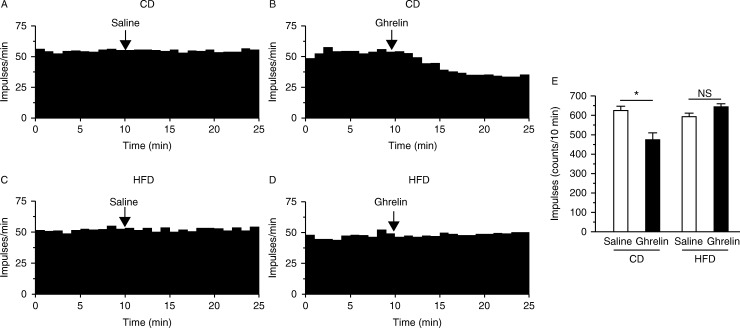
Electrophysiological effect of ghrelin on gastric vagal afferent activity in CD- or HFD-fed mice. Representative data for gastric vagal afferent discharge rates are shown in A, B, C and D. Gastric vagal afferent discharge in CD-fed mice was not affected by s.c. administration of saline (A), whereas it was inhibited by administration of ghrelin (B). Gastric vagal afferent discharge in HFD-fed mice was not affected by saline (C) or ghrelin (D). (E) Ghrelin significantly attenuated impulses 10 min after its injection in CD- but not HFD-fed mice. *P<0.05 versus CD-fed mice subjected to saline injection. NS, not significant.

A representative record of the vagal afferent electrical activity in response to saline or ghrelin administration is shown in Fig. 3. Ghrelin attenuated the vagal afferent nerve activity in CD- but not HFD-fed mice (Fig. 3A, B, C and D). Ghrelin-induced suppression of the number of spikes was abrogated in HFD-fed mice (Fig. 3E).

### Fos expression

**Figure 4:**
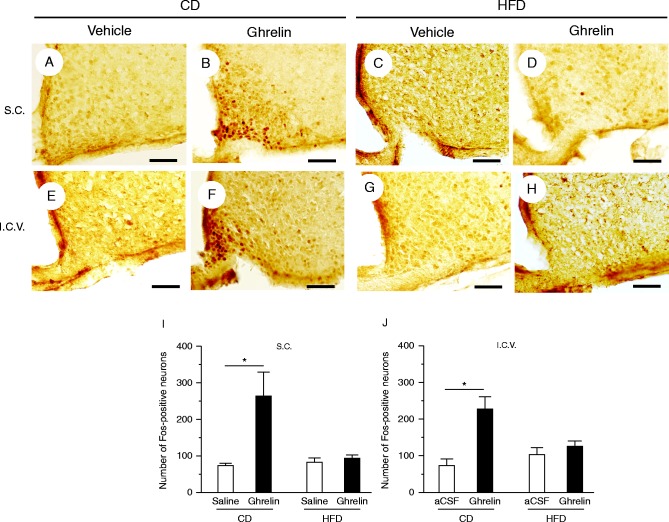
Representative Fos expression patterns in hypothalamic arcuate nucleus in response to s.c. administration of saline (vehicle) (A) or ghrelin (B) in CD-fed mice and saline (C) or ghrelin (D) in HFD-fed mice. Fos expression patterns in the arcuate nucleus in response to i.c.v. administration of artificial cerebrospinal fluid (aCSF; vehicle) (E) or ghrelin (F) in CD-fed mice and aCSF (G) or ghrelin (H) in HFD-fed mice. Numbers of Fos-immunoreactive neurons of mice subjected to s.c. (I) or i.c.v. administration (J) of ghrelin or vehicle. Values are means±s.e.m. *P<0.05 versus saline or aCSF. Scale bars, 50 μm.

Both s.c. and i.c.v. administrations of ghrelin caused a significant increase in the numbers of Fos-immunoreactive neurons in the hypothalamic arcuate nucleus of CD- but not HFD-fed mice (Fig. 4).

### *Ghsr* mRNA expression

**Figure 5:**
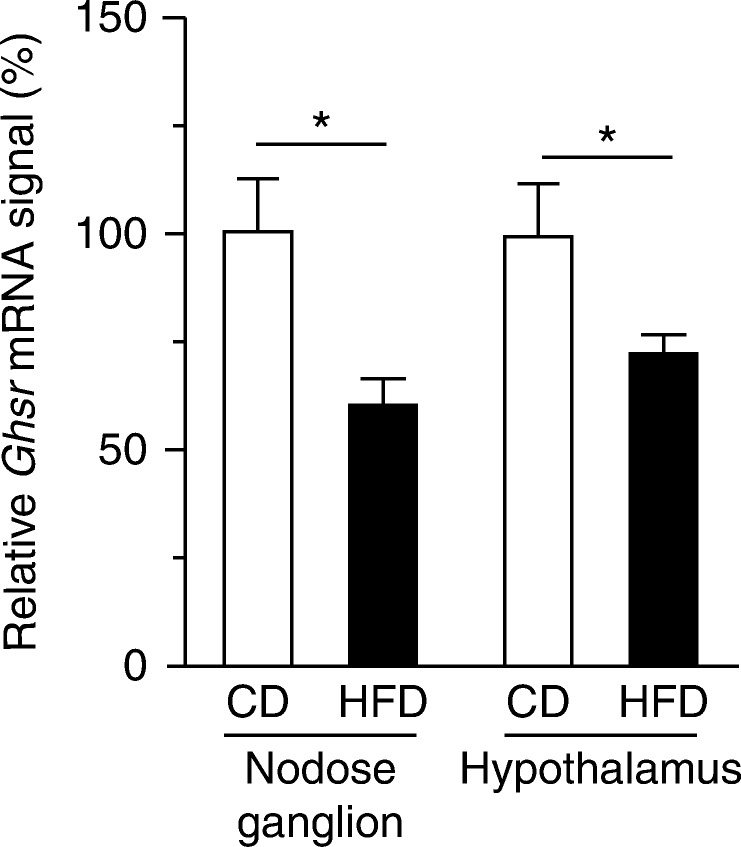
mRNA expressions of *Ghsr* in the nodose ganglion and hypothalamus of CD- or HFD-fed mice. Values are means±s.e.m. *P<0.05 versus CD.

In HFD-fed mice, the *Ghsr* mRNA levels in the nodose ganglion and hypothalamus were significantly lower than those in CD-fed mice (Fig. 5).

### Inflammatory mRNA and immunohistochemistry

**Figure 6:**
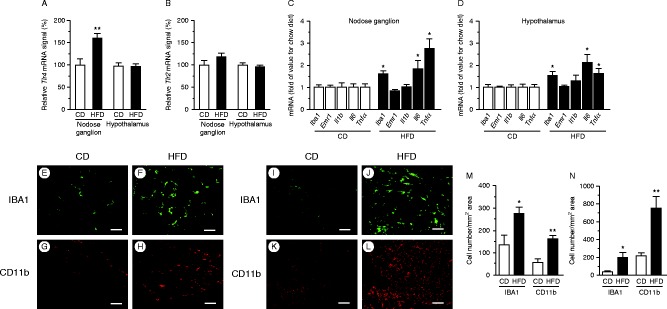
mRNA expression of *T* *lr4* (A) and *Tlr2* (B) in the nodose ganglion and hypothalamus of CD- or HFD-fed mice. Expressions of genes encoding macrophage markers (*Iba1* and *Emr1*) and inflammatory cytokines (*Il1b*, *Il6*, and *Tnf* *α*) in the nodose ganglion (C) and hypothalamus (D) of CD- or HFD-fed mice. mRNAs were quantitated relative to *Gapdh* or *Tbp* housekeeping genes, and relative levels are presented as fold change relative to CD. Values are means±s.e.m. *P<0.05 and **P<0.01 versus CD. Histochemical analyses of HFD-induced macrophage accumulation in the nodose ganglion and hypothalamus. Immunohistochemical detection of IBA1 (E and F) and CD11b (G and H) in the nodose ganglion and IBA1 (I and J) and CD11b (K and L) in the hypothalamus of CD- or HFD-fed mice. Numbers of cells stained with IBA1 or CD11b antibody in the nodose ganglion (M) and hypothalamus (N). Values are means±s.e.m. *P<0.05 and **P<0.01 versus CD. Scale bars, 50 μm.

**Figure 7:**
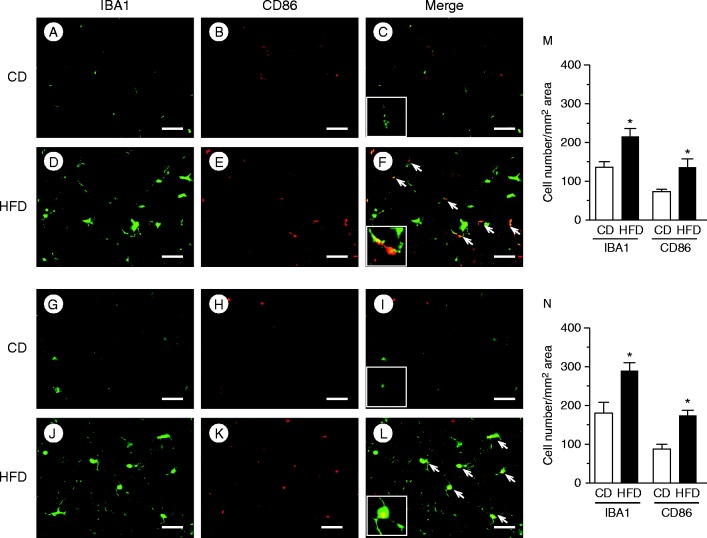
Immunohistochemical analyses of HFD-induced M1 macrophage accumulation in the nodose ganglion and hypothalamus. IBA1, CD86, and merged images of the nodose ganglion (A, B, C, D, E and F), and the hypothalamus (G, H, I, J, K and L) of CD- or HFD-fed mice. Arrows indicate co-localization of CD86 with IBA1. The insets in C, F, I and L are higher magnification examples of IBA1^+^/CD86^+^ cells. Numbers of cells stained with IBA1 or CD86 antibody in the nodose ganglion (M) and hypothalamus (N). Values are means±s.e.m. *P<0.05 versus CD. Scale bars, 50 μm.

TLR4 expression was significantly higher in HFD-fed mice than in CD-fed mice in the nodose ganglion, but not in the hypothalamus (Fig. 6A). In both groups, we observed no significant difference in the expression of TLR2 in the nodose ganglion or hypothalamus (Fig. 6B). The *Iba1*, *Il6*, and *Tnf* *α* mRNAs were significantly upregulated in the nodose ganglion in HFD-fed mice relative to CD-fed mice (Fig. 6C). Hypothalamic expressions of mRNAs of *Iba1*, *Il6*, and *Tnf* *α* were also significantly upregulated in HFD-fed mice relative to CD-fed mice (Fig. 6D). The numbers of macrophages stained with anti-IBA1 (Fig. 6E and F) or anti-CD11b (Fig. 6G and H) antibodies in the nodose ganglion, as well as those stained with anti-IBA1 (Fig. 6I and J) or anti-CD11b (Fig. 6K and L) antibodies in the hypothalamus of HFD-fed mice, were significantly higher than those in CD-fed mice (Fig. 6M and N). Expression levels of the M1 macrophage markers IBA1 and CD86 in the nodose ganglion (Fig. 7A, B, D and E) and hypothalamus (Fig. 7G, H, J and K) of HFD-fed mice were significantly higher than those in CD-fed mice (Fig. 7M and N). Approximately 30% of IBA1-positive macrophages/microglia expressed CD86 immunoreactivity both in the nodose ganglion (Fig. 7C and F) and hypothalamus (Fig. 7I and L) of HFD-fed mice.

### Effects of ghrelin on phosphorylations of ERK1/2 and AMPKα

**Figure 8:**
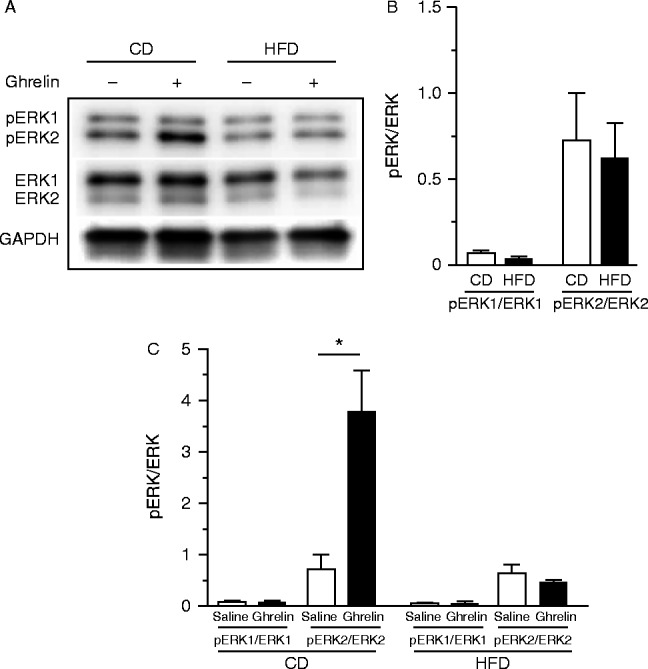
Representative western blots for pERK1/2 and ERK1/2 in the nodose ganglia of CD- and HFD-fed mice (A). Levels of basal phosphorylation of ERK1 and ERK2 in the nodose ganglion of CD- and HFD-fed mice (B). Phosphorylated ERK1 and ERK2 in the nodose ganglion after s.c. administration of ghrelin (C). GAPDH was used as a control. Values are means±s.e.m. and represent the ratio of the intensity of bands corresponding to pERK1 and ERK1 or pERK2 and ERK2. *P<0.05 vs saline.

ERK1/2 and pERK1/2 were detected in the nodose ganglia of both CD- and HFD-fed mice (Fig. 8A). HFD did not affect the phosphorylation of either ERK1 or ERK2, normalized against the corresponding total ERK level (Fig. 8B). Ghrelin administration significantly increased pERK2 in CD- but not HFD-fed mice (Fig. 8C).

**Figure 9:**
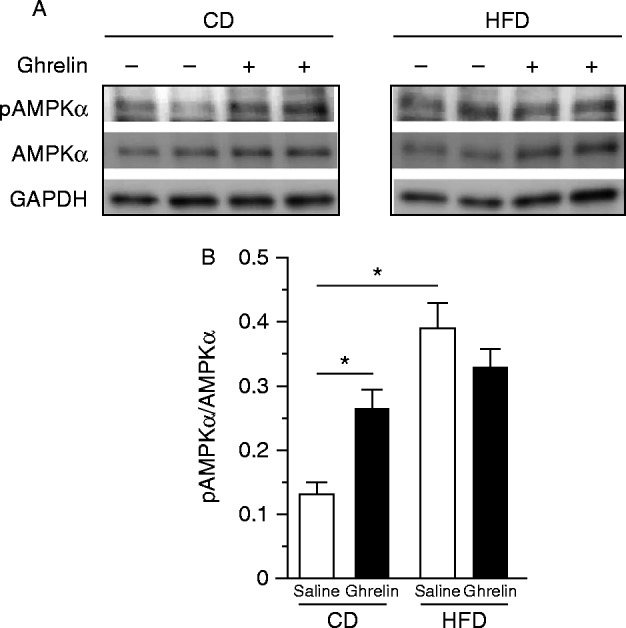
Representative western blots for pAMPKα and AMPKα in the nodose ganglia of CD- or HFD-fed mice (A). Ghrelin promoted phosphorylation of AMPKα in CD- but not in HFD-fed mice (B). GAPDH was used as a control. Values are means±s.e.m. and represent the ratio of the intensity of bands corresponding to pAMPK and AMPK. *P<0.05 versus saline.

The basal level of AMPKα in the nodose ganglion of HFD-fed mice was significantly higher than that in CD-fed mice (Fig. 9A and B). Ghrelin administration significantly increased pAMPKα in CD- but not HFD-fed mice (Fig. 9B).

## Discussion

In this study, we showed that peripheral ghrelin resistance is associated with inflammation in the nodose ganglia of HFD-fed mice, resulting in an impairment of the vagal afferent system. Results of previous studies indicated that both peripheral and central administration of ghrelin were unable to stimulate food intake in HFD-fed mice (Perreault *et al*. 2004, Briggs *et al*. 2010, Gardiner *et al*. 2010). Here, we confirmed these findings in mice given 12-week HFD, in which 60% of the energy was provided as fat. Moreover, s.c. administration of ghrelin did not evoke suppression of vagal afferent activity, phosphorylation of ERK2 and AMPKα in the nodose ganglion, or Fos expression in the hypothalamic arcuate nucleus. We also showed that neutralization of ghrelin by the i.c.v. administration of anti-ghrelin IgG failed to suppress natural feeding in DIO mice, indicating that endogenous ghrelin did not act as an orexigenic peptide under HFD. A HFD appeared to induce central ghrelin resistance by reducing both *Ghsr* expression in the hypothalamus and NPY/AgRP neuronal responsiveness to ghrelin (Briggs *et al*. 2010). Based on these findings, along with the upregulation of ghrelin secretion upon fasting and downregulation of its secretion after meals, ghrelin is considered not to promote obesity, but rather to prevent starvation (Andrews *et al*. 2010, McFarlane *et al*. 2014). In this study, the disappearance of plasma ghrelin after its administration to HFD-fed mice was similar to that in CD-fed mice, indicating that the pharmacokinetics of ghrelin in DIO mice may not account for ghrelin resistance. The vagal afferent nerve is the major pathway conveying ghrelin's signals for starvation to the brain (Date *et al*. 2002). We postulated that downregulation of *Ghsr* expression in the nodose ganglion of DIO mice could blunt transmission of gastric-derived ghrelin's signals.

Several lines of evidence indicated that HFD activates an inflammatory response in the systemic organs and hypothalamus of rodents and humans (De Souza *et al*. 2005, Milanski *et al*. 2009, Posey *et al*. 2009). Hypothalamic inflammation induced by a HFD-manifested neuronal injury triggers a reactive gliosis by microglia and astrocytes (Thaler *et al*. 2012). These cellular responses occur selectively in the hypothalamic arcuate nucleus, a target region of gastric-derived ghrelin's signals. We postulated that HFD also caused inflammatory changes in the nodose ganglion. The calcium-binding protein IBA1 is a marker of microglia/macrophage activation in the nervous system (Ito *et al*. 1998). CD11b is another marker of microglia/macrophage activation/recruitment (Perego *et al*. 2011). In this study, HFD induced macrophage activation and inflammatory responses in the nodose ganglion, as assessed by the increased numbers of IBA1- and CD11b-positive macrophages, and production of inflammatory cytokines such as *Iba1*, *Il6*, and *Tnf* *α*. CD86, an activating and costimulatory protein, is expressed in activated microglia (Henkel *et al*. 2006). We also found that abundant IBA1^+^ microglia expressed CD86, a marker of M1 macrophage/microglia. They were morphologically rounded and more ramified, indicating that more activated subtypes of macrophages/microglia were present. Several lines of evidence indicate that the vagal afferent nerve transmits gut-derived inflammatory signals to the brain (Goehler *et al*. 1999, Hosoi *et al*. 2005). In the present study, as we found *Tlr4* expression was increased in the nodose ganglion of HFD-fed mice, further investigation is needed to determine whether HFD-induced TLR4 activation in the gut transmits inflammatory signals to the nodose ganglion via the vagal afferent.

The reported mechanisms by which ghrelin exerts its biological activities are complex. Ghrelin activates MAPKs, including ERK1/2 (Mousseaux *et al*. 2006). ERK1/2 are protein–serine/threonine kinases involved in the activation of nuclear transcription factors controlling proliferation, differentiation, and cell death (Gutkind 2000). In our hands, ghrelin administration to CD-fed mice, but not HFD-fed mice, induced ERK2 phosphorylation in the nodose ganglion. The reduced expression of the *Ghsr* in DIO mice could result in the downregulation of the GHSR-mediated ERK-signaling pathway in the vagal afferent system.

AMPK is a key regulatory enzyme in cellular energy balance. Changes in hypothalamic AMPK activity regulate food intake (Minokoshi *et al*. 2004), and ghrelin activates hypothalamic AMPK (Kola *et al*. 2005). In this study, administration of ghrelin to CD-fed mice induced AMPK phosphorylation in the nodose ganglion, but had no effect in DIO mice. Basal AMPK phosphorylation in the nodose ganglia of HFD-fed mice was significantly higher than that of CD-fed mice. The higher basal AMPK phosphorylation in skeletal muscle of DIO mice is thought to contribute to leptin resistance (Martin *et al*. 2006). The pathophysiological significance of altered AMPK phosphorylation level and AMPK's role as the ghrelin signaling molecule in the nodose ganglion should be investigated in future work.

Briggs *et al*. (2014) recently showed that 3-week HFD-induced hyperleptinemia (approximately 7 ng/ml) can cause ghrelin resistance. Furthermore, they observed hypothalamic gliosis, as revealed by increases in the numbers of glial fibrillary acidic protein-positive glia and their projections. They explained the ghrelin resistance under 3-week HFD as a consequence of leptin's counteracting effect against ghrelin on hypothalamic NPY/AgRP neurons. We detected marked hyperleptinemia (89 ng/ml) of HFD-fed mice in this study, in which leptin did not exert an effect as an anorectic protein, as reported in many investigations (El-Haschimi *et al*. 2000, Zhang *et al*. 2008). These mice exhibited leptin resistance, indicating that hyperleptinemia did not contribute to ghrelin resistance. We thought that long-term HFD causes ghrelin resistance via chronic inflammation in the nodose ganglion and hypothalamus.

Biosynthesis of ghrelin is downregulated in obesity, and fasting plasma ghrelin concentrations in humans are negatively correlated with body weight, percentage body fat, and fat mass (Tschöp *et al*. 2001, Shiiya *et al*. 2002). Notably, results of one study indicated that there was no difference in basal ghrelin levels between lean and obese individuals, and that ghrelin increased energy intake in mildly obese humans (Druce *et al*. 2005). We fed mice a HFD (60% calorie from fat) for 12 weeks and they become severely obese. Ghrelin did not stimulate feeding in these obese mice. In contrast, ghrelin increased food intake during the early stage (1–2 weeks) of HFD (23.5% calorie from fat) treatment in mildly obese mice (Briggs *et al*. 2014). There may be a possibility that the duration of fat exposure and the degree of obesity could affect ghrelin sensitivity in humans and animals.

Ghrelin modulates immune processes, in part, by suppressing sympathetic nerve activity and by reducing inflammatory cytokine production in activated macrophages (Dixit *et al*. 2004). The vagal efferent nerve activity was reduced in obese patients, and selective cholinergic activation of the vagal efferent nerve in DIO mice suppressed obesity-related inflammation and restored metabolic complications (Pavlov & Tracey 2012). Berkseth *et al*. (2014) recently showed that 4-week CD (12 kcal from fat) following 16-week HFD (60% kcal from fat) given to C57BL/6J mice reversed hypothalamic inflammation. A future study may delineate whether inflammation in the nodose ganglion and ghrelin resistance caused by HFD are also reversible after switching to a low-fat diet.

In conclusion, this study offers the new insights that HFD causes inflammatory responses in the nodose ganglion in addition to the hypothalamus. Additionally, ghrelin resistance in obese states could be associated with inflammation in the nodose ganglion. The vagal afferent nerve may act as a novel pathway that regulates the peripheral inflammatory signal to the brain.

## Author contribution statement

F N, K T, H S, and M N designed the experiments; F N, K T, T M Z W, C N, and A S M M performed the experiments; F N, K T, and C N analyzed the data. All authors prepared and approved the final version of the manuscript.

## Figures and Tables

**Table 1:** Characteristics and blood parameters of mice fed a CD or a HFD. Data are expressed as means±s.e.m. (*n*=6–8)

	**CD**	**HFD**
Initial body weight (g)	23.9±0.6	24.2±0.6
Final body weight (g)	30.2±0.4	49.6±0.9^‡^
Epididymal fat weight (g)	0.54±0.01	2.59±0.24^‡^
24-h food intake (g)	3.27±0.07	2.62±0.04^‡^
24-h energy intake (kJ)	46.5±1.0	57.4±0.8^‡^
Blood glucose (mmol/l)	6.5±0.3	9.8±0.7*
Plasma insulin (ng/ml)	0.35±0.01	1.65±0.09^‡^
Plasma leptin (ng/ml)	6.1±0.4	89.1±5.6^‡^
Plasma ghrelin (fmol/ml)	65.7±9.9	8.8±1.2^†^
Plasma des-acyl ghrelin (fmol/ml)	1255±157	937±54*

*P<0.05, ^†^ *P*<0.01, and ^‡^ *P*<0.001 versus CD.
